# Corrigendum: Contribution of classical end-joining to PTEN inactivation in p53-mediated glioblastoma formation and drug-resistant survival

**DOI:** 10.1038/ncomms15795

**Published:** 2017-08-14

**Authors:** Youn-Jung Kang, Barbara Balter, Eva Csizmadia, Brian Haas, Himanshu Sharma, Roderick Bronson, Catherine T Yan

Nature Communications 8 Article: 14013 ; DOI: 10.1038/ncomms14013 (2017); Published 01 17 2017; Updated 08 14 2017

‘In the References section of this Article, the citation listed as reference 17 is incorrect, and should have referred to the following paper:

Forbes, S. A. *et al.* COSMIC: exploring the world's knowledge of somatic mutations in human cancer. *Nucleic Acids Res*. **43**(Database issue): D805-D811 (2015).’

