# Supplementary material for: Contribution of classical end-joining to PTEN inactivation in p53-mediated glioblastoma formation and drug-resistant survival
Source: Nat Commun. 2017 Jan 17;8:14013. doi: 10.1038/ncomms14013 (PMC5247582; doi:10.1038/ncomms14013)
Supplement: Supplementary Information — Supplementary figures and supplementary tables. [file ncomms14013-s1.pdf]

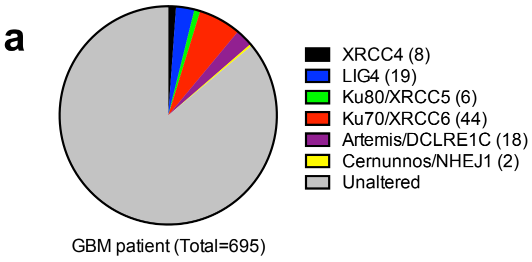

b

GeneA	GeneB	*P*-Value	Log Odds Ratio	Association
TP53	PTEN	9.25E-06	1.0856	Tendency towards co-occurrence (Significant)
TP53	XRCC4	0.0208	Infinity	Tendency towards co-occurrence (Significant)

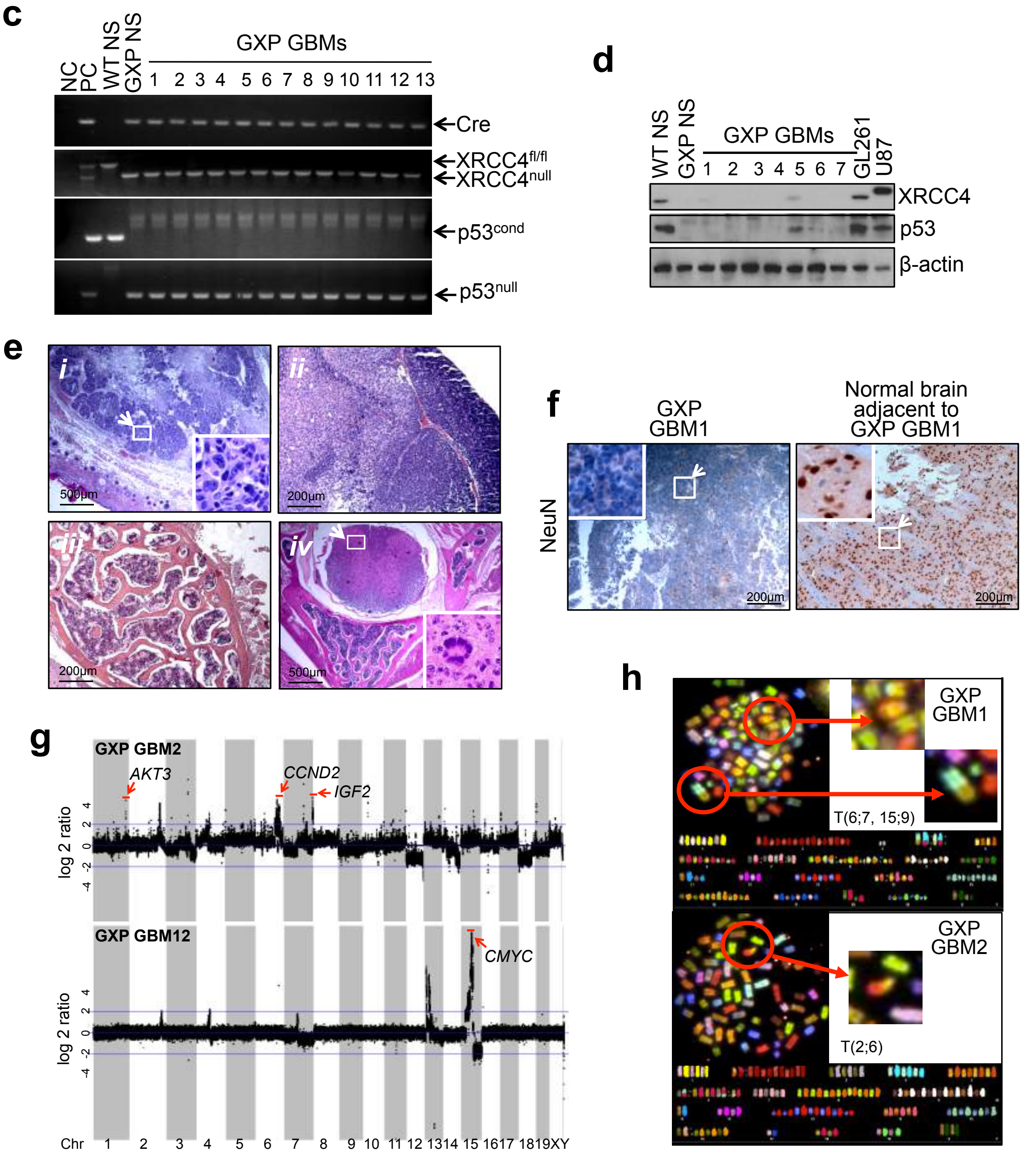

Supplementary Figure 1: Characterisation of GBMs arose from hGFAP-Cre-XRCC4$^{fl/fl}$p53$^{fl/fl}$ mice, Related to Figure 1.

(a) A pie chart representing the number of GBM patients (*n*=97/695) with NHEJ gene defects (COSMIC database). **(b)** A table summarising the tendency for co-occurrence of molecular alterations in TP53 with PTEN or XRCC4 in human GBMs (cBioPortal database). **(c)** Total genomic DNA from GXP mutant mouse brain tissues was isolated and PCR amplified for sequences specific for Cre (200bp), XRCC4$^{fl/fl}$ (1125bp), XRCC4^{null} (862bp), p53^{cond} (584bp), and p53^{null} (431bp) alleles. The positions of these bands are indicated by arrows on the right. Lane 1: Negative Control (NC), no DNA, Lane 2: Positive Control (PC) for each sequence, Lane 3: DNA from WT (XP-undeleted) neural stem cells, Lane 4: DNA from GXP neural stem cells, and Lane 5-17: DNA from GXP GBMs. **(d)** Immunoblot analysis of XRCC4 and p53 in 7 independent GXP GBMs compared to WT NS, GXP NS, GL261, or U87. **(e)** H&E of medulloblastomas observed in the spinal cord (***i***) and brain (***ii***) of GXP mice, and high-grade astrocytic gliomas observed in the spinal cord of GXP mice those are also harboring GBMs in their brains (***iii*** and ***iv***)**.** Boxed regions indicated with white arrows in (***i***) and (***iv***) are shown at higher magnification and enlarged. **(f)** Immunohistochemical staining in GXP GBM1 compared to its adjacent normal brain tissue with antibody against NeuN. Boxed regions indicated with an arrow in each image are shown at higher magnification and enlarged. **(g)** Array comparative genomic hybridization (aCGH) plots of whole spectrum of chromosomes (X-axis) and log_2 ratio (Y-axis) are shown for two representative GXP GBMs (#2 and #12 are shown). Amplified regions containing *AKT3* (chr1), *CCND2* (chr6), and *IGF2* (chr7) in GXP GBM2 (upper panel), and *CMYC* (chr15) in GXP GBM12 (bottom panel)

are indicated. **(H)** Spectral karyotype analyses displaying high levels of aneuploid and non-recurrent translocations in GXP GBM1 (translocations in chromosome 6, 7, 9, and 15) and GXP GBM2 (translocation in chromosome 2 and 6).

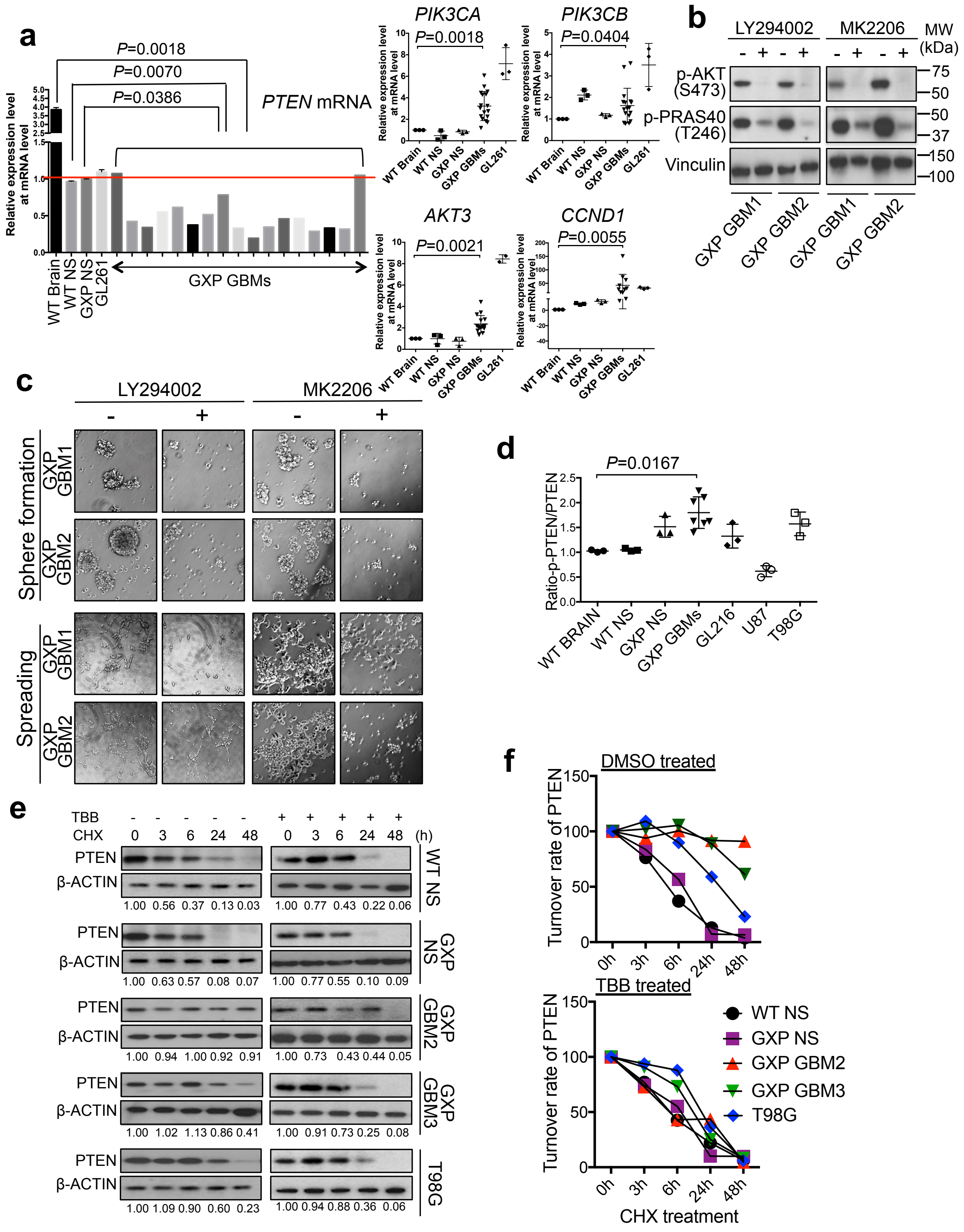
a
P=0.0018
P=0.0070
P=0.0386
PTEN mRNA
Relative expression level at mRNA level
WT Brain
WT NS
GXP NS
GL261
GXP GBMs
PIK3CA
P=0.0018
PIK3CB
P=0.0404
AKT3
P=0.0021
CCND1
P=0.0055
WT Brain
WT NS
GXP NS
GXP GBMs
GL261
b
LY294002
MK2206
MW (kDa)
p-AKT (S473)
p-PRAS40 (T246)
Vinculin
75
50
50
37
150
100
GXP GBM1
GXP GBM2
GXP GBM1
GXP GBM2
c
LY294002
MK2206
Sphere formation
Spreading
GXP GBM1
GXP GBM2
d
P=0.0167
Ratio-p-PTEN/PTEN
WT BRAIN
WT NS
GXP NS
GXP GBMs
GL216
U87
T98G
e
TBB
CHX
0 3 6 24 48 (h)
PTEN
β-ACTIN
1.00 0.56 0.37 0.13 0.03
1.00 0.77 0.43 0.22 0.06
WT NS
1.00 0.63 0.57 0.08 0.07
1.00 0.77 0.55 0.10 0.09
GXP NS
1.00 0.94 1.00 0.92 0.91
1.00 0.73 0.43 0.44 0.05
GXP GBM2
1.00 1.02 1.13 0.86 0.41
1.00 0.91 0.73 0.25 0.08
GXP GBM3
1.00 1.09 0.90 0.60 0.23
1.00 0.94 0.88 0.36 0.06
T98G
f
DMSO treated
TBB treated
Turnover rate of PTEN
0h
3h
6h
24h
48h
CHX treatment
WT NS
GXP NS
GXP GBM2
GXP GBM3
T98G

Supplementary Figure 2: GXP GBMs display downregulated *PTEN* mRNA and stabilised PTEN protein leading to hyperactivation of the downstream PI3K-AKT signaling pathway, Related to Figure 2.

(a) Real-time reverse transcriptase PCR (RT-PCR) was used to quantitate the mRNA expression and validate the results from RNA-seq analysis of *PTEN*, PI3K-AKT related genes (*PIK3CA*, *PIK3CB*, *AKT3*, and *CCND1*) in 17 independent GXP GBMs compared to WT adult forebrain, WT or GXP NS, and GL261. RT-PCR results were analysed using the Mann Whitney test including a *P*-value in each graph. Data are representative of three biological replicates. All graphs depict mean±s.e.m. **(b)** Efficacy of LY294002 or MK2206 in GXP GBM1 and 2 was measured by immunoblot analysis of p-AKT (S473) and p-PRAS40 (T246). Vinculin was used for loading control. **(c)** Impact of LY294002 or MK2206 on the growth of GXP GBM1 and 2 was displayed by showing morphological alterations (sphere formation and cell spreading). Cells were treated with LY294002 (25uM) or MK2206 (2uM) for 48h in a suspension culture for sphere formation or on the matrigel-coated cover glass for cell spreading, respectively. **(d)** Densitometry of immunoblot bands from 3 independent experiments shown in Fig. 2d was measured using Image J. The graph presents the ratio of p-PTEN to the total PTEN (Y-axis) in GXP GBMs compared to WT adult brain, WT or GXP NS, GBM cell lines (murine GL261, human U87, or human T98G) (X-axis). The statistical analysis for the comparison between the ratio in WT adult brain and GXP GBMs was performed using the Mann Whitney test including a *P*-value. Results are pooled from three independent experiments. All graphs depict mean±s.e.m. **(e)** Immunoblot analysis of PTEN after cyclohexamide (200μg/ml) treatment for an indicated time period in GXP GBMs

compared to WT or GXP NS, and T98G cell line with DMSO or TBB treatment. Numbers below each blot are corresponding to the densitometry of each band, which was normalised to the β-ACTIN and then standardised to the PTEN expression level at 0h of cyclohexamide. **(f)** Two graphs summarising and comparing the turnover rate of PTEN protein (Y-axis) for indicated time period of cyclohexamide (X-axis) in DMSO-treated (upper) or TBB-treated (bottom) GXP GBMs compared to WT or GXP NS, and T98G cell line.

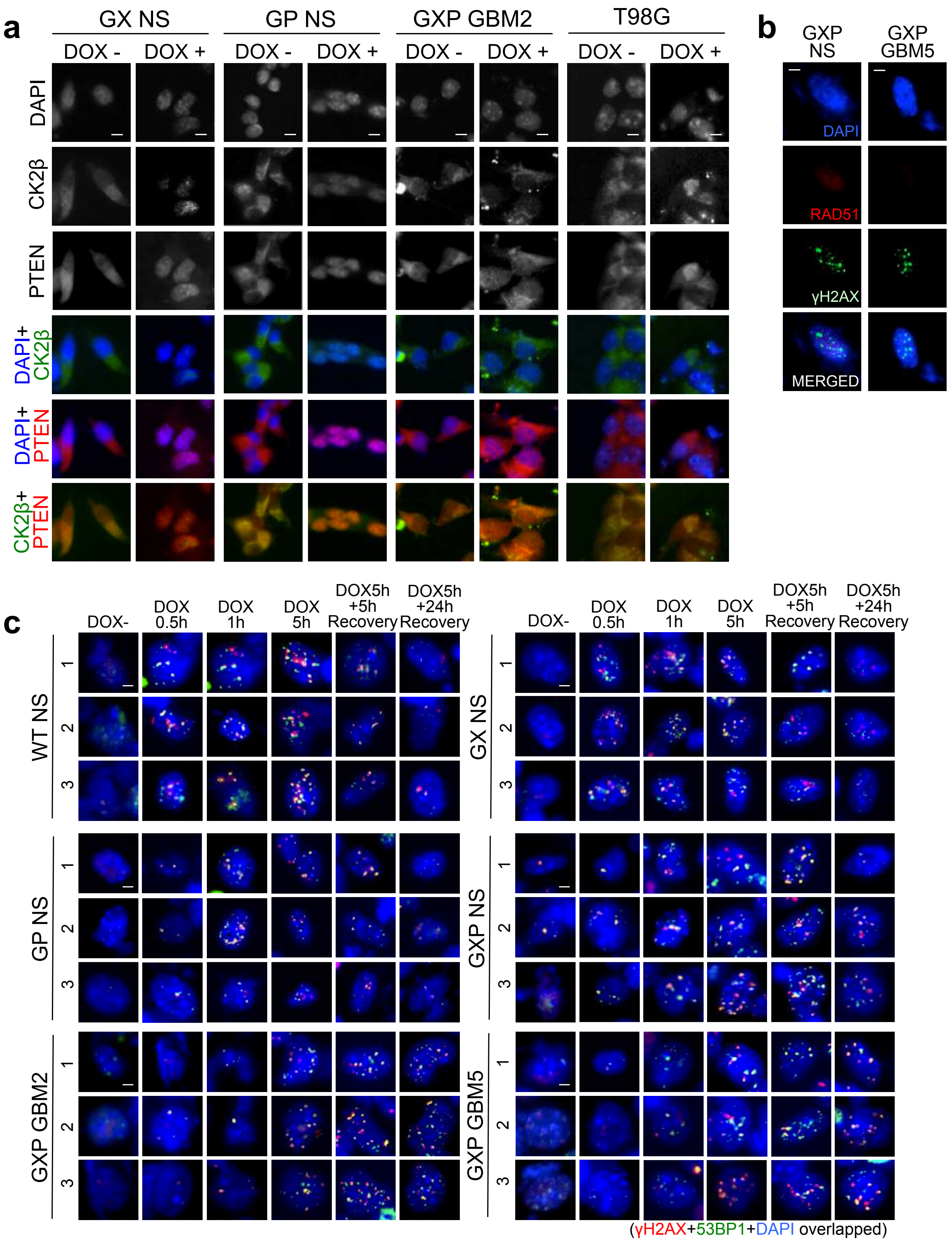

a
GX NS
GP NS
GXP GBM2
T98G
DOX -
DOX +
DAPI
CK2β
PTEN
DAPI+ CK2β
DAPI+ PTEN
CK2β+ PTEN
b
GXP NS
GXP GBM5
DAPI
RAD51
γH2AX
MERGED
c
DOX-
DOX 0.5h
DOX 1h
DOX 5h
DOX5h +5h Recovery
DOX5h +24h Recovery
WT NS
GX NS
GP NS
GXP NS
GXP GBM2
GXP GBM5
1
2
3
(γH2AX+53BP1+DAPI overlapped)

Supplementary Figure 3: DNA damage-induced nuclear PTEN distribution is attenuated in both murine and human GBMs, Related to Figure 3.

(a) Co-Immunofluorescence (IF) staining of CK2β and PTEN displaying distribution of CK2β or PTEN with or without doxorubicin (DOX) treatment (0.5μM for 5h) in GX NS, GP NS, GXP GBM2, and T98G. Nuclei were visualised by DAPI staining. IF images shown are representative of the majority of the cells for each condition. Scale bar: 10μm. **(b)** Co-IF staining of DOX-induced foci of γH2AX and RAD51 in GXP NS and GXP GBM 5. Cells were exposed to DOX (0.5μM) for 5h prior to fixation, and antibody and DAPI nuclear staining. Scale bar: 10μm. **(c)** Co-IF staining of DOX-induced foci of γH2AX and 53BP1 in WT NS, GX NS, GP NS, GXP NS, and GXP GBM2 & 5. Cells were exposed to DOX (0.5μM) for 0h, 0.5h, 1h or 5h, and allowed to recover for 5h or 24h prior to fixation, and antibody and DAPI nuclear staining. Three representative images for each condition are shown. Scale bar: 10μm.

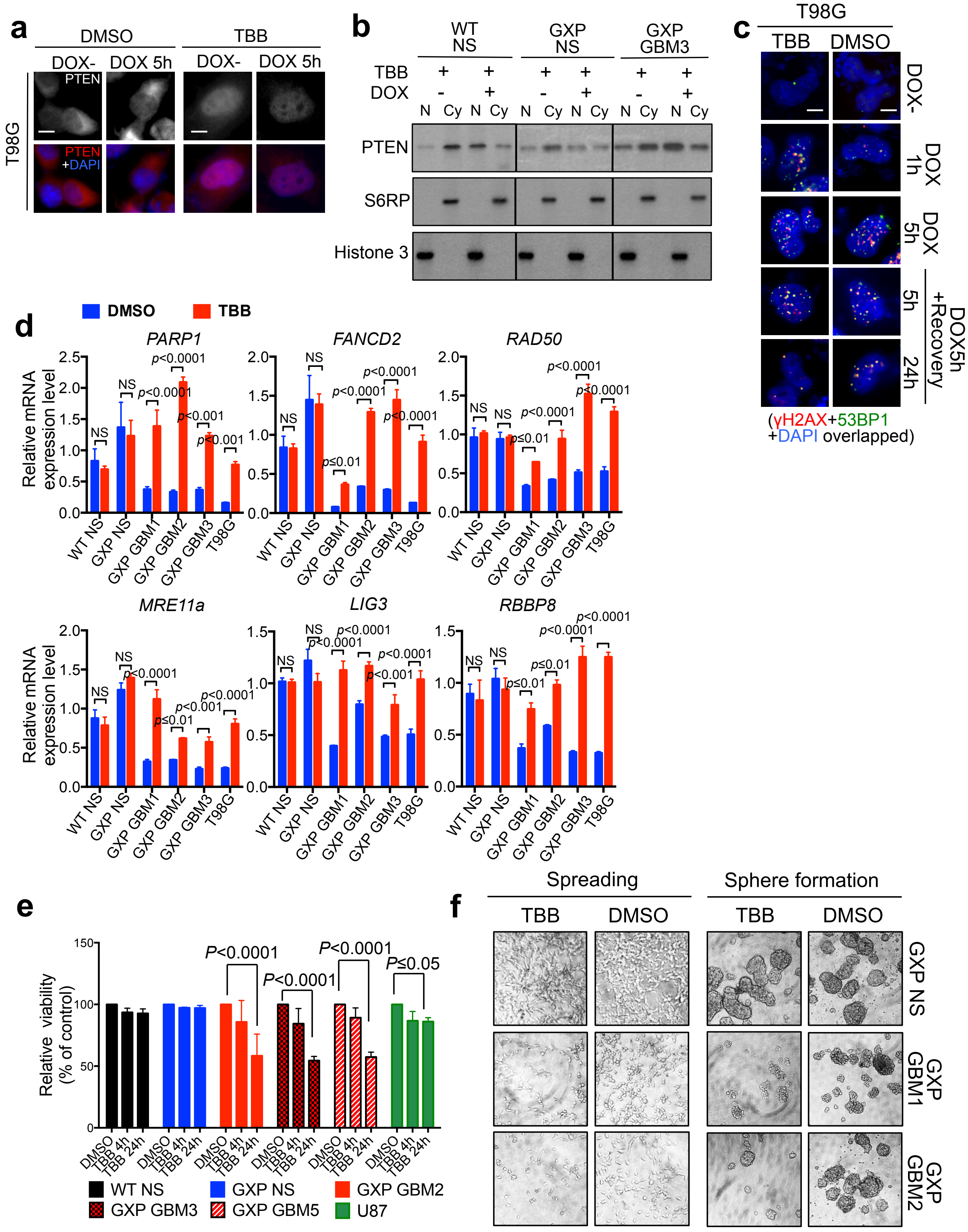

a
DMSO
TBB
DOX-
DOX 5h
T98G
PTEN
PTEN
+DAPI
b
WT NS
GXP NS
GXP GBM3
TBB
DOX
N
Cy
PTEN
S6RP
Histone 3
c
T98G
TBB
DMSO
DOX-
DOX 1h
DOX 5h
DOX5h +Recovery
5h
24h
(γH2AX+53BP1 +DAPI overlapped)
d
DMSO
TBB
PARP1
FANCD2
RAD50
MRE11a
LIG3
RBBP8
Relative mRNA expression level
NS
p<0.0001
p<0.001
p≤0.01
p≤0.05
WT NS
GXP NS
GXP GBM1
GXP GBM2
GXP GBM3
T98G
e
Relative viability (% of control)
P<0.0001
P≤0.05
DMSO
TBB 4h
TBB 24h
WT NS
GXP NS
GXP GBM2
GXP GBM3
GXP GBM5
U87
f
Spreading
Sphere formation
TBB
DMSO
GXP NS
GXP GBM1
GXP GBM2

Supplementary Figure 4: CK2β-mediated DNA damage response and repair functions underlie the tumorigenicity in GXP GBMs, Related to Figure 4.

(a) IF staining of PTEN with DAPI nuclear staining in DMSO or TBB (25μM for 24h)-treated T98G cells in the absence of DOX or after 5h of exposure to DOX (0.5μM). DOX was added 5h prior to the end point of DMSO or TBB treatment. Representative images are shown. Scale bar: 10μm. **(b)** Immunoblot analysis of nuclear (N) and cytosolic (Cy) distributions of PTEN in TBB-treated (25μM/24h) WT or GXP NS, or GXP GBM3 in DOX- (DMSO-treated) and DOX+ (0.5μM/5h) conditions. Loading controls: S6RP (cytosolic) and Histone3 (nuclear). **(c)** Co-IF of γH2AX and 53BP1 foci formation with DAPI nuclear staining in DMSO or TBB (25μM for 24h)-treated T98G with DOX- (0h), or 1h or 5h of DOX (0.5μM) treatment followed by 5h or 24h of recovery time. **(d)** RT-PCR was used to quantitate the mRNA expression of HR or A-EJ-related genes (*PARP1*, *FANCD2*, *RAD50*, *MRE11A*, *LIG3*, and *RBBP8*) in 3 independent GXP GBMs compared to WT or GXP NS, or T98G with DMSO or TBB treatment. RT-PCR results were analysed using the Two-way ANOVA test with Tukey's multiple comparisons test including *P*-values in each graph. Results are pooled from three independent experiments. All graphs depict mean±s.e.m. **(e)** Relative viability of cells from WT NS, GXP NS, 3 independent GXP GBMs, and U87 in response to TBB treatment (25μM) for 48h. Cell viability results were analysed using the Two-way ANOVA test with Tukey's multiple comparisons test. *P*-values are indicated in each graph. Results are pooled from three independent experiments. All graphs depict mean±s.e.m. **(f)** Impact of TBB treatment on cell growth in 2 independent GXP GBMs compared to GXP NS was

displayed by presenting morphological alterations in sphere formation and spreading on matrigel. Cells grown in a suspension culture or on matrigel-coated cover glass were treated with TBB (25μM) or DMSO for 48h.

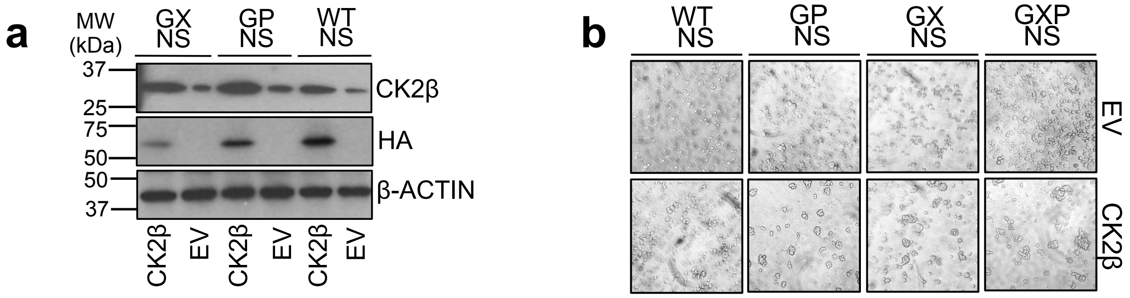

Supplementary Figure 5: CK2β overexpression promotes cellular transformation of GXP NS, Related to Figure 5.

(a) Efficacy of HA-tagged lentiviral cDNA overexpression of CK2β in WT, GX, or GP NS was measured by immunoblot analysis of CK2β. Effects of CK2β overexpression in WT, GX, GP and GXP NS in liquid cultures are shown in **(b).**

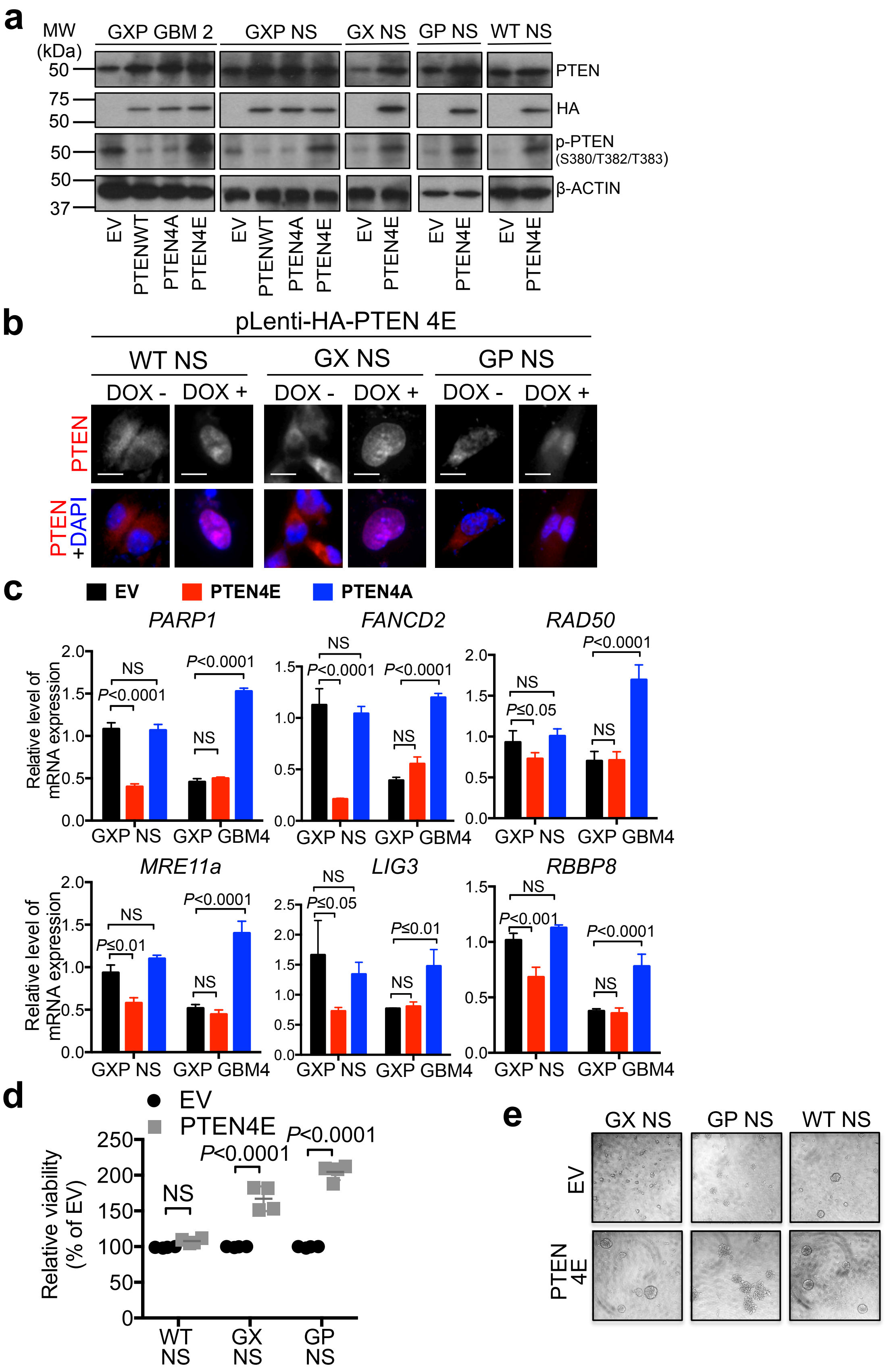

a
MW (kDa)
GXP GBM 2
GXP NS
GX NS
GP NS
WT NS
PTEN
HA
p-PTEN (S380/T382/T383)
β-ACTIN
EV
PTENWT
PTEN4A
PTEN4E
b
pLenti-HA-PTEN 4E
DOX -
DOX +
PTEN
PTEN +DAPI
c
EV
PTEN4E
PTEN4A
PARP1
FANCD2
RAD50
MRE11a
LIG3
RBBP8
Relative level of mRNA expression
GXP NS
GXP GBM4
NS
P<0.0001
P≤0.05
P≤0.01
P<0.001
d
EV
PTEN4E
Relative viability (% of EV)
WT NS
GX NS
GP NS
e
GX NS
GP NS
WT NS
EV
PTEN 4E

Supplementary Figure 6: Attenuated PTEN-mediated DNA damage signaling underlies GBM tumorigenicity, Related to Figure 6.

(a) Efficacy of HA-tagged lentiviral cDNA overexpression of PTEN (WT, 4A, or 4E) in WT, GX, GP, or GXP NS, or GXP GBM2 was measured by immunoblot analysis of total PTEN and phospho-PTEN (S380/T382/T383). **(b)** IF staining of PTEN in pLenti-HA-PTEN4E-transduced WT, GX, or GP NS with or without exposure to DOX (0.5μM/5h). Nuclei were visualised by DAPI staining. IF images shown are representatives of majority of cells in each condition. **(c)** RT-PCR quantitating the mRNA expression of HR or A-EJ-related genes (*PARP1*, *FANCD2*, *RAD50*, *MRE11A*, *LIG3*, and *RBBP8*) in EV, PTEN4E, or 4A-transduced GXP GBM4 compared to GXP NS with each transduction. RT-PCR results were analysed using the Two-way ANOVA test with Tukey's multiple comparisons test. *P*-values are indicated in each graph. Results are pooled from three independent experiments. All graphs depict mean±s.e.m. **(d)** Relative cell viability of EV or PTEN4E-transduced WT, GX, or GP NS. Data from 3 independent experiments were normalized to the value of EV-transduced cells in each group and analysed using the two-way ANOVA with Tukey's multiple comparisons test including *P*-values in each graph. Data are representative of three biological replicates. All graphs depict mean±s.e.m. **(e)** Efficacy of overexpression of PTEN4E in WT, GX, or GP NS was assessed in sphere formation in liquid cultures compared to control vector (EV).

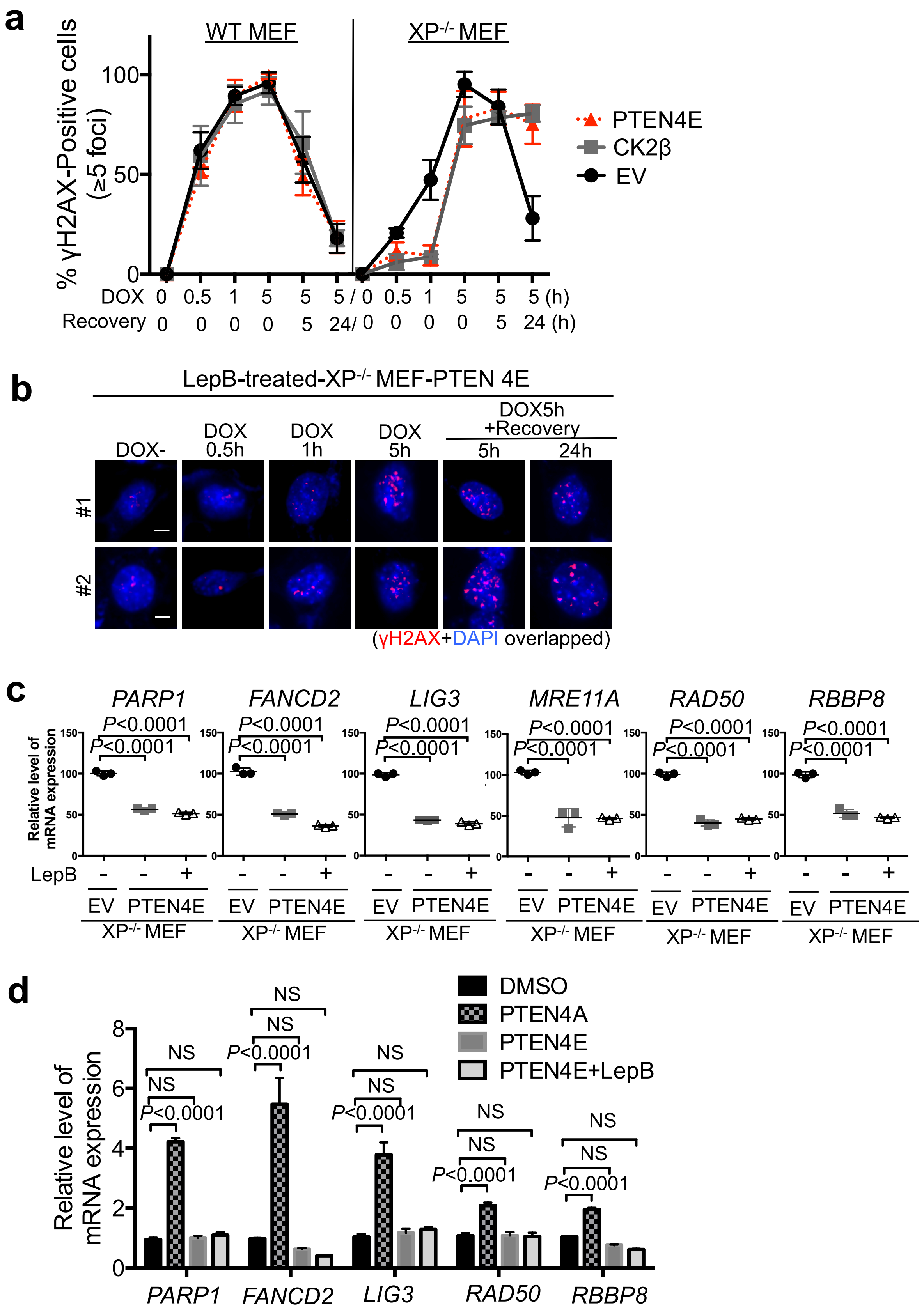
a
WT MEF
XP-/- MEF
% γH2AX-Positive cells (≥5 foci)
100
50
0
PTEN4E
CK2β
EV
DOX 0 0.5 1 5 5 5 / 0 0.5 1 5 5 5 (h)
Recovery 0 0 0 0 5 24/ 0 0 0 0 5 24 (h)
b
LepB-treated-XP-/- MEF-PTEN 4E
DOX-
DOX 0.5h
DOX 1h
DOX 5h
DOX5h +Recovery
5h
24h
#1
#2
(γH2AX+DAPI overlapped)
c
PARP1
FANCD2
LIG3
MRE11A
RAD50
RBBP8
P<0.0001
Relative level of mRNA expression
LepB
EV
PTEN4E
XP-/- MEF
d
DMSO
PTEN4A
PTEN4E
PTEN4E+LepB
NS
P<0.0001
Relative level of mRNA expression
PARP1
FANCD2
LIG3
RAD50
RBBP8

Supplementary Figure 7: Aberrant cytosolic distribution of inactive PTEN attenuates DNA damage response and repair regulating its conformational status, Related to Figure 7.

(a) A graph quantifying the percent of γH2AX-positive PTEN4E, CK2β, or EV-transduced WT or $XP^{-/-}$ MEF cells (Y-axis) at indicated time points of DOX treatment (X-axis). Representative 50 cells were randomly selected for quantification from Fig. 7b. Cells with ≥5 foci were considered as γH2AX-positive. **(b)** IF staining of DNA damage induced γH2AX foci with DAPI nuclear staining in Leptomycin B (LepB)-treated-PTEN4E-transduced $XP^{-/-}$ MEF cells: DOX- and after 0.5h, 1h or 5h of DOX (0.5μM) exposure, followed by 5h or 24h of recovery time. Two representative images for each condition are shown. Scale bar: 10μm. **(c)** RT-PCR quantitating the mRNA expression of the HR or A-EJ-related genes (*PARP1*, *FANCD2*, *RAD50*, *MRE11A*, *LIG3*, and *RBBP8*) in LepB-treated PTEN4E-transduced $XP^{-/-}$ MEF cells compared to untreated-PTEN4E or EV-transduced $XP^{-/-}$ MEF cells. Normalised RT-PCR results were analysed using one-way ANOVA with Tukey's multiple comparisons test with *P*-values. Results are pooled from three independent experiments. All graphs depict mean±s.e.m. **(d)** RT-PCR quantitating the mRNA expression of the HR or A-EJ-related genes (*PARP1*, *FANCD2*, *RAD50*, *MRE11A*, *LIG3*, and *RBBP8*) in DMSO, PTEN4A, 4E, or LepB treated-PTEN4E-transduced T98G. RT-PCR results were analysed using the Two-way ANOVA test with Tukey's multiple comparisons test. *P*-values are indicated in each graph.

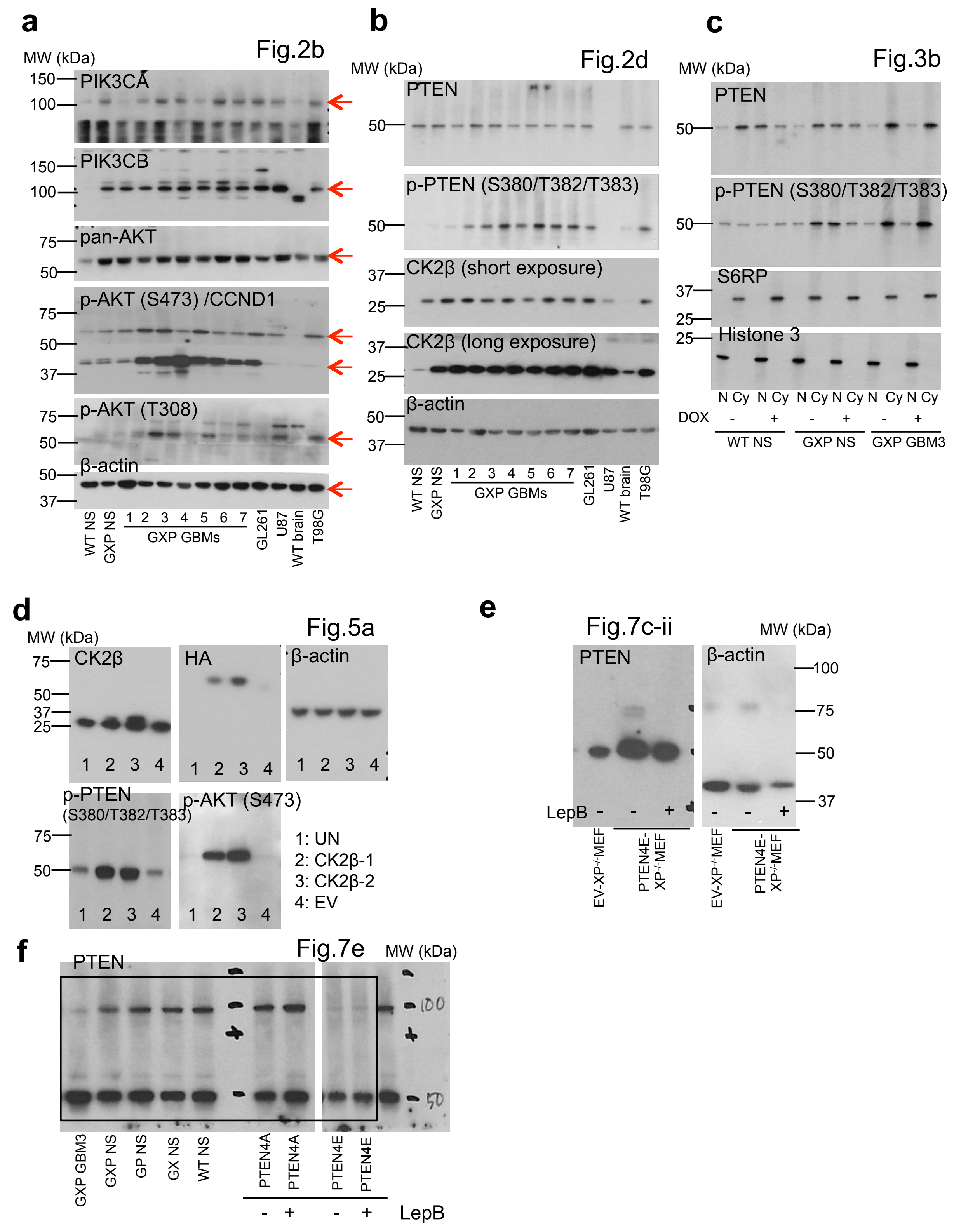

Supplementary Figure 8: Original immunoblots for indicated figures.

NHEJ gene	Patient sample number$	TP53	PTEN	RB1	NF1	CDKN2A	CDKN2B	CDKN2C	EGFR	PIK3CA	AKT3	NOTCH2	BRCA2	RAD50	MYC	MET	PDGFRA
XRCC4	TCGA-FG-8189-01																
	TCGA-DU-A6S6-01																
	TCGA-HT-8107-01																
	TCGA-FG-A4MX-01																
	TCGA-FG-A713-01																
	TCGA-P5-A5F6-01																
	TCGA-DU-7292-01																
	TCGA-14-0871-01				nonsense									del			
LIG4	TCGA-15-0742-01					del	del		amp	del	amp	missense					
	TCGA-14-0871-01																
	TCGA-14-0787-01					del	del		amp								
	TCGA-06-0168-01		frameshift														
	TCGA-DU-7304-02																
	TCGA-VM-A8C9-01																
	TCGA-12-5295-01					del	del		amp								
	TCGA-FG-A6IZ-01																
	TCGA-FG-5964-01																
	TCGA-19-2625-01	missense	missense	frameshift					amp								
	TCGA-QH-A65X-01																
	TCGA-DH-A669-02																
	TCGA-HT-A4DS-01																
	TCGA-28-2499-01																
	TCGA-P5-A72W-01																
	TCGA-FG-A6J1-01																
	TCGA-27-2526-01		missense						amp								
	TCGA-27-2521-01	frameshift				del	del						missense		amp		
	TCGA-FG-A6J3-01																
Ku80/XRCC5	TCGA-FG-A711-01																
	TCGA-P5-A5F6-01																
	TCGA-HT-A74J-01																
	TCGA-DH-A7UT-01																
	TCGA-P5-A72U-01																
	TCGA-QH-A6CV-01																
Ku70/XRCC6	TCGA-P5-A5F6-01																
	TCGA-DU-6404-01																
	TCGA-P5-A72U-01																
	TCGA-TM-A84B-01																
	TCGA-27-1835-01	missense															
	TCGA-DU-A5TT-01																
	TCGA-HT-A617-01																
	TCGA-CS-6186-01																
	TCGA-HT-7860-01																
	TCGA-DU-8161-01																
	TCGA-19-2625-01	missense	missense	frameshift					amp								
	TCGA-FG-A4MY-01																
	TCGA-E1-A7YU-01																
	TCGA-19-2620-01				spilice	del	del		amp								
	TCGA-QH-A6CS-01																
	TCGA-FG-A4MX-01																
	TCGA-DU-A5TY-01																
	TCGA-06-0747-01																
	TCGA-VM-A8CD-01																
	TCGA-06-0158-01					del	del		amp								
	TCGA-19-1389-02																
	TCGA-DU-8158-01																
	TCGA-06-2562-01				frameshift	del											
	TCGA-E1-A7YE-01																
	TCGA-DB-A64O-01																
	TCGA-E1-A7YJ-01																
	TCGA-DU-6404-02																
	TCGA-41-4097-01		missense		nonsense	del	del										
	TCGA-15-0742-01					del	del		amp	del	amp	missense					
	TCGA-06-0744-01	missense							amp								
	TCGA-06-0141-01																
	TCGA-S9-A89V-01																
	TCGA-HW-A5KK-01																
	TCGA-S9-A7R7-01																
	TCGA-06-1804-01		missense						amp								
	TCGA-06-0221-02	frameshift															
	TCGA-26-5132-01			nonsense					amp								
	TCGA-14-1034-01																
	TCGA-06-5418-01		missense			del	del										
	TCGA-DU-5847-01																
	TCGA-27-2519-01	frameshift	missense	del													
	TCGA-06-0878-01					del	del		amp								
	TCGA-DU-A76L-01																
	TCGA-DB-5273-01																
Artemis/DCLRE1C	TCGA-12-3652-01					del	del		amp								
	TCGA-26-5133-01	missense			frameshift										amp		
	TCGA-76-4932-01																
	TCGA-28-5216-01	missense	missense			del	del	del								amp	amp
	TCGA-28-5209-01		missense			del	del		amp								
	TCGA-41-2571-01		nonsesne														
	TCGA-41-2572-01								amp								
	TCGA-76-4929-01	missense				del	del										
	TCGA-19-2625-01	missense	missense	frameshift													
	TCGA-28-2499-01																
	TCGA-FG-8189-01																
	TCGA-12-3650-01					del	del		amp								
	TCGA-28-2513-01		splice			del	del		amp								
	TCGA-12-5295-01					del	del		amp								
	TCGA-14-0871-01	frameshift			nonsense									del			
	TCGA-19-2629-01	splice				del	del										
	TCGA-14-1825-01	nonsense	del			del	del										
	TCGA-32-4213-01		frameshift	frameshift		del	del		amp								
Cernunnos/NHEJ1	TCGA-FG-A4MX-01																
	TCGA-DU-A5TT-01																

$Co-occurrence of molecular alterations of NHEJ genes with well-known tumor suppressors or oncogenes has been analyzed for only accessible GBM patient cohort (*n*=32).

Supplementary Table 1. **GBM patients with defective NHEJ gene expression level.**

A list of GBM patients (*n*=97/695) including reduced NHEJ gene expression level, displaying co-occurrence of molecular alterations of in GBM-relevant tumour suppressors or oncogenes (from COSMIC and cBioPortal database)

# of samples with cut-off (copy number (log value)-0.25)*	Chromosome	Start	End	Cytoband	# of genes in peak (tumor)$	Known cancer genes in the region	Genes with gene expression (mRNA level) correlated with copy number
**Amplification**							
6	6	126,276,673	127,276,754	qF3	59	*CCND2*	*CCND2*
6	7	151,229,117	152,524,553	qF5	14	*CCND1*	*CCND1*
5	1	176,725,424	177,264,825	qH4	3	*AKT3*	*AKT3*
5	6	17,366,685	17,675,432	qB4 - qB5	2	*MET*	*MET*
3	3	31,112,701	33,509,723	qA1 - qB	14	*PIK3CA*	*PIK3CA*
5	7	149,554,071	149,888,993	qF5	10	*IGF2*	-
3	10	117,126,160	117,147,559	qD2	4	*MDM2*	
3	5	2,973,996	5,906,471	qA1	22	*CDK6*	*CDK6*
3	11	7,376,728	52,634,468	qA2	60	*EGFR*	*EGFR*
3	15	61,015,472	71,475,856	qA1.1	11	*CMYC*	*CMYC*
2	5	72,059,152	76,183,767	qC3.1 - qE3	34	*PDGFRA*	*PDGFRA*
2	10	126,500,617	126,504,379	qD2 - qD3	184	*CDK4*	
2	1	134,885,866	134,921,147	qD - qE4	814	*MDM4*	
1	12	10,954,690	13,687,088	qA1.1	5	*MYCN*	*MYCN*
**Deletion**							
5	4	whole chromosome		qC4 - qC5	8	*CDKN2A*	-
5	4	whole chromosome		qC4 - qC5	8	*CDKN2B*	-
4	19	30,912,135	37,472,148	qC1	25	*PTEN*	*PTEN*
4	4	whole chromosome		qC6 - qD2.2	282	*CDKN2C*	-

* For amplifications, number of samples with copy number gain at each locus; for deletions, number of samples with copy number loss. (Out of 17 samples.)

$ The highest number of genes within amplicons or deleted regions for each sample is noted.

⌘ Frequencies of amplifications or deletions of well-established cancer genes (TCGA, 2008; Verhaak et al., 2010) were obtained from the critical analyses using the Integrative Genomic Viewer (IGV) and NEXUS (Li and Olivier, 2013; Pena-Llopis and Brugarolas, 2013).

Supplementary Table 2. Frequencies of copy number alterations of well-known cancer genes identified in XRCC4/p53-deficient murine GBMs[⌘] using the Integrative Genomic Viewer (IGV) and NEXUS.

Cre	CGTATAGCCGAAATTGCCAG
	CAAAACAGGTAGTTATTCGG
XRCC4	GTCCAAGGAGGTGGCCACTAGT
	GATGTCACCTCAATGATGCTGGT
	TCAGCCATGCTAGCAAGCACC
$p53^{cond}$	AAGGGGTATGAGGGACAAGG
	GAAGACAGAAAAGGGGAGGG
$p53^{null}$	ACCGCTTCCTCGTGCTTTAC
	ATGGGAGGCTGCCAGTCCTACCC

Supplementary Table 3. A list of the primer sequences used for genotyping PCR assays

PTEN	Forward	GATAGCATTTGCAGTATAGAGCGTGCAGATA
	Reverse	GATCTTCATCAAAAGGTTCATTCTCTGGATCAG
PIK3CA	Forward	CCACGACCATCTTCGGGTG
	Reverse	ACGGAGGCATTCTAAAGTCACTA
PIK3CB	Forward	TTCTTTTCAGTGTTGTGACCAAG
	Reverse	GCCCCGAATGTGGTAAGTT
AKT3	Forward	CATAGGCTATAAGGAGAAACC
	Reverse	TTGGATAGCTTCCGTCCAC
CCND1	Forward	TTTCTTTCCAGAGTCATCAAGTGTG
	Reverse	ACCAGCCTCTTCCTCCACTTC
MRE11A	Forward	CCTCTTATCCGACTACGGGTG
	Reverse	ACTGCTTTACGAGGTCTTCTACT
RAD50	Forward	TGATAAGTTGTCTTGGGGTTTCC
	Reverse	CTGTGTCTGACGCACCTGT
LIG3	Forward	TGCCTGAAAAAGGTACTGTTGG
	Reverse	ATGCCACAAAGTAGCGTTTGA
PARP1	Forward	GGTCTTTAAGAGCGACGCTTAT
	Reverse	TTCTGTGTCTTGACCATGCAC
FANCD2	Forward	GGCTGAACATAAGGCTTTGGA
	Reverse	AATGTGTAGAGAACGCACTGTC

Supplementary Table 4. A list of the primer sequence pairs used for quantitative RT-PCR based analysis of mRNA expression
